# Seventy years since the invention of the averaging technique in Neurophysiology: Tribute to George Duncan Dawson

**DOI:** 10.1590/0004-282X-ANP-2021-0263

**Published:** 2021-12-07

**Authors:** Otto Jesus Hernández Fustes, Cláudia Suemi Kamoi Kay, Paulo José Lorenzoni, Renata Dal-Prá Ducci, Lineu Cesar Werneck, Rosana Herminia Scola

**Affiliations:** 1 Universidade Federal do Paraná Complexo Hospital de Clínicas Departamento de Clínica Médica Curitiba PR Brazil Universidade Federal do Paraná, Complexo Hospital de Clínicas, Departamento de Clínica Médica, Serviço de Neurologia, Serviço de Doenças Neuromusculares e Desmielinizantes, Curitiba PR, Brazil.

**Keywords:** Evoked Potentials, Neurology, Neurophysiology, Somatosensory, Potenciais Evocados, Neurologia, Neurofisiologia, Potenciais Somatossensoriais Evocados

## Abstract

In 1951, the physiologist George Duncan Dawson presented his work with the averaging of the signal in the evoked potentials (EPs), opening a new stage in the development of clinical neurophysiology. The authors present aspects of Professor Dawson’s biography and a review of his work on the EPs and, mainly, the article reveals the new technique in detail that would allow the growth of the clinical application of the visual, auditory, and somatosensory EPs.

## RESUMO

Em 1951 o fisiologista George Duncan Dawson apresentou seu trabalho com a promediação de sinal nos potenciais evocados, abrindo uma nova etapa no desenvolvimento da neurofisiologia clínica. Os autores apresentam aspectos da biografia do professor Dawson e uma revisão de seus trabalhos sobre os potenciais evocados, principalmente do artigo que mostrava a nova técnica, que viria a permitir o crescimento da aplicação clínica dos potenciais evocados visual, auditivo e somatossensitivo.

The initial description of the evoked potential (EP) by Richard Caton in 1875 was when he observed on the galvanometer fluctuations in the electrical activity of the monkey’s exposed cortex in response to the stimulation of his lips and the light shone in one eye, and it took more than half a century, due to the lack of adequate equipment to record these evoked responses, even the pioneering work of the English physiologist George Duncan Dawson^1^.

EPs recorded on the human’s scalp were difficult to extract due to the high voltage of the cortical oscillating electroencephalogram noise, among other causes. Recording of EP is a noninvasive method for investigating brain activity. This tool corresponds to voltage fluctuations blocked by time derived from the continuous electroencephalography (EEG) signal in response to a specific peripheral sensor^2^.

Dawson demonstrated that electrical stimulation of a peripheral nerve in human produces brain responses^3^, and the analysis of these responses at the time, distally stimulating the median or ulnar nerves, they generated a low amplitude action potential^4^, making their study difficult, a problem that was solved with the summation technique he developed.

**Figure 1:**
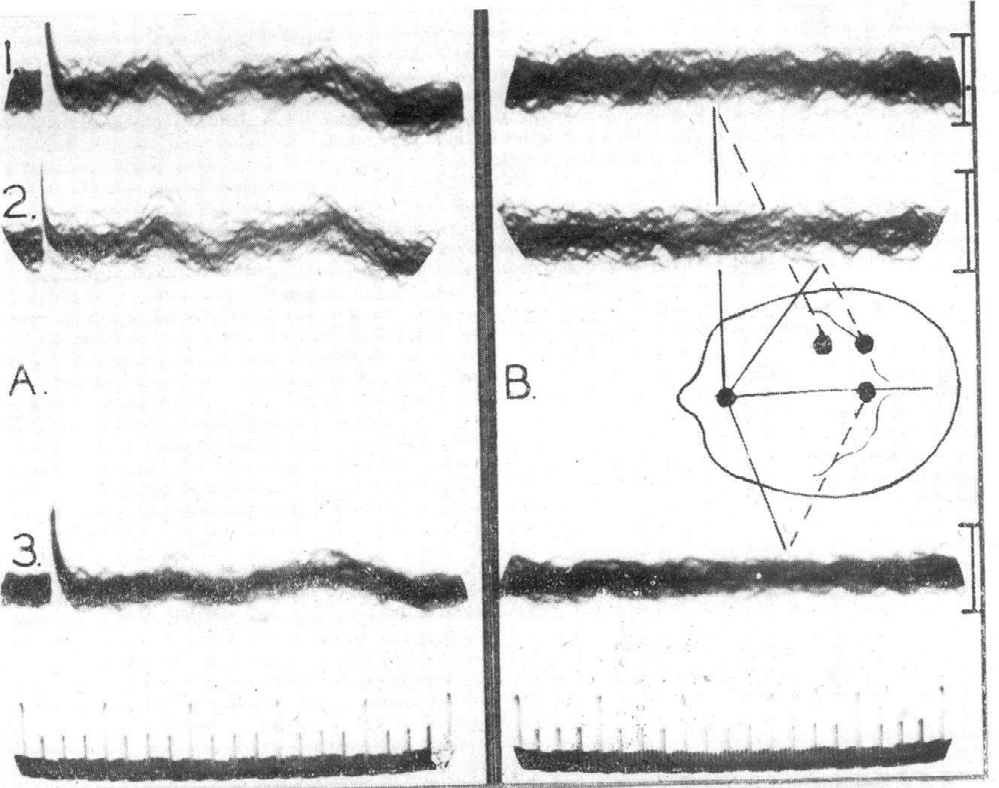
Dawson photograph of the superimposed responses to 50 successive stimulations of the ulnar nerve at the wrist (with permission of BMJ Publishing Group Ltd., License Number: 5061900358457).

In 1947, George Dawson blocked the stimulation of the median nerve for scanning his oscilloscope. Pairs of electrodes placed on the human’s scalp, overlying the parietal lobes, captured responses of repetitions of electrical stimuli of the median nerves which were amplified and displayed on a storage oscilloscope screen. Multiple Dawson’s photographs overlapping somatosensory evoked responses showed a first positive peak of latency at 28 ms^3^ (Figure 1). Cerebral action potentials evoked by stimulation of somatic nerves may be detected on the human’s scalp by superimposing many records. In 1970, Jewett and Williston used Dawson’s averaging technique to record the minute auditory response of electrodes on the human’s scalp^1^.

On May 19, 1951, at the Physiological Society in Cambridge, Dawson demonstrated his equipment built with his assistant Jack Pitman, using a bench of 25 condensers and a moving paper camera motor^5,6^. In 1954, Dawson improved the equipment by developing the electromechanical signal mediator (EMSM), a motorized rotary switch that powers each individual scalp response blocked by a single median nerve stimulus in the time of 1 of the 62 capacitors every 0.1 s. The EMSM discharged each capacitor in series into an amplifier connected to a storage oscilloscope^7^.

Dawson’s contribution to the advancement of EP registration was the solution he found to solve the signal-to-noise problem in obtaining EP. For that, it established the method of the first sum and average, that is, the responses evoked at each repetition of the stimuli were displayed on an oscilloscope and superimposed on a photographic film. Thus, brain activity blocked by time resulted in overexposure in part of the film, while random activity exposed the entire film only slightly. This method of photographic film over the trace, therefore, made it possible to suppress unrelated spontaneous potentials, extracting the phases of significantly low amplitude response evoked by stimulation^5^.

Due to the impact of his work, George Duncan Dawson is considered the father of EP studies and from then on, EP became an independent field of neurophysiology. The averaging method was subsequently improved to bring the current computerized digital averaging methods that allow multiple responses to be averaged, and the old galvanometers were progressively replaced by highly sensitive amplifiers.

## GEORGE DUNCAN DAWSON

**Figure 2:**
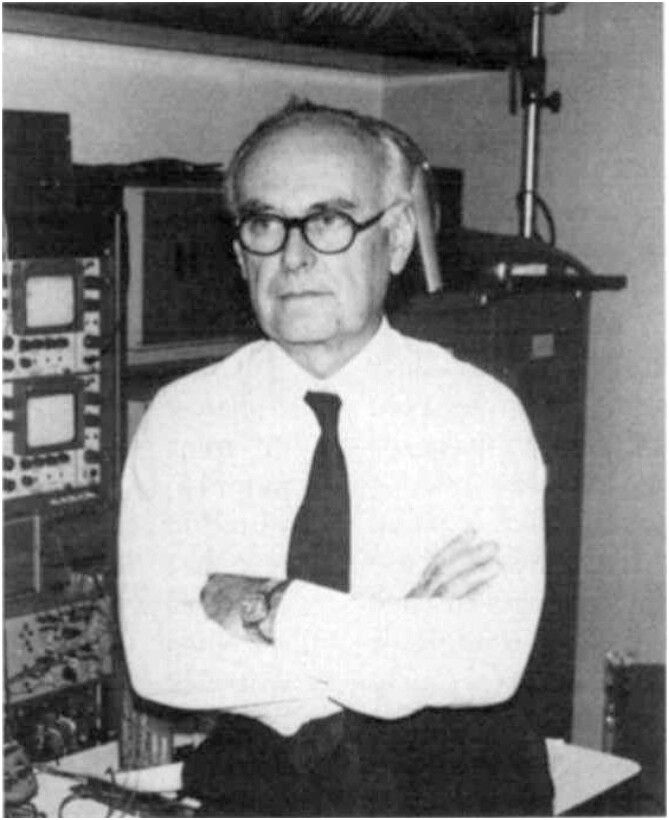
George Duncan Dawson^7^.

George Duncan Dawson (Figure 2) was born in Manchester in 1912, and his father was a pathologist at the Manchester Royal Infirmary. Dawson was educated at Repton and Manchester University, graduating in medicine in 1936, 3 years before he completed his master’s degree in research on nerve action potentials. In 1938, at the Manchester Royal Infirmary, he helped set up Professor Sir Geoffrey Jefferson’s EEG laboratory, later dedicated to the study of brain electrical activity in patients with epilepsy at the David Lewis Colony in Sandlebridge, Cheshire^6^. Together with Gray Walter, he published two articles considered classic in EEG.^8,9^ Although Dawson was really interested in the potential of EEG as a scientific tool, he had already investigated a special group of epileptics in whom myoclonic reflexes were associated with large isolated waves in the EEG.

In 1944, Dawson was part of the Medical Research Council Unit of the National Hospital for Nervous Diseases, Queen Square, under the guidance of Professor Carmichael, where he developed techniques to identify small electroencephalographic signals against background noise while studying patients with epilepsy. At that time, he studied a patient with myoclonic seizures that could be caused by light electrical stimuli applied to a peripheral device nerve. The resulting cortical waves were large enough (50 μV) to be captured with the naked eye, from the spontaneous EEG background on the oscilloscope^10^. It was the first description of the potential giants.

Dawson became the head of the Department of Clinical Neurophysiology at the London Institute of Psychiatry in 1961, was named the Second Professor of Physiology at University College London in 1966, where he remained until his retirement, and continued his research work on epilepsy in the Lingfield Epileptic Colony, developing the computer-based methods to evaluate treatments with antiepileptic drugs. Dawson died on November 13, 1983, and his work endures as a milestone in the development of Clinical Neurophysiology^6^.

In conclusion, Professor Dawson was an emeritus professor of Physiology at University College London. The signal averaging technique, originally described by Dawson, has been improved with the development of computerized processing. The technique now applies a stimulus repeatedly, records the evoked response in the corresponding area of the brain, and mathematically calculates the average change in the number of stimuli and responses.

Dawson’s contribution to Neurophysiology deserved recognition for the highest order, including a Nobel Prize, a task that Franklin F. Offner was trying to promote at the time of his death. However, the researcher’s modesty prevented him from taking any action designed to bring him greater fame, “it would be the furthest thing from your mind, and it was totally foreign to his nature”^7^.
